# Molecular subtype-specific responses of colon cancer cells to the SMAC mimetic Birinapant

**DOI:** 10.1038/s41419-020-03232-z

**Published:** 2020-11-30

**Authors:** Michael Fichtner, Emir Bozkurt, Manuela Salvucci, Christopher McCann, Katherine A. McAllister, Luise Halang, Heiko Düssmann, Sinéad Kinsella, Nyree Crawford, Tamas Sessler, Daniel B. Longley, Jochen H. M. Prehn

**Affiliations:** 1 Department of Physiology and Medical Physics, Centre for Systems Medicine, Royal College of Surgeons in Ireland, Dublin, Ireland; 2 Centre for Cancer Research and Cell Biology, Queen’s University Belfast, Belfast, UK; 3 Present Address: Department of Genetics and Bioengineering, Faculty of Engineering, Izmir University of Economics, Balcova, Izmir Turkey; 4 Present Address: Program in Immunology, Clinical Research Division, Fred Hutchinson Cancer Research Center, Seattle, WA USA

**Keywords:** Colon cancer, Apoptosis, Preclinical research

## Abstract

Colorectal cancer is a molecularly heterogeneous disease. Responses to genotoxic chemotherapy in the adjuvant or palliative setting vary greatly between patients, and colorectal cancer cells often resist chemotherapy by evading apoptosis. Antagonists of an inhibitor of apoptosis proteins (IAPs) can restore defective apoptosis signaling by degrading cIAP1 and cIAP2 proteins and by inhibition of XIAP. Due to the multiple molecular mechanisms-of-action of these targets, responses to IAP antagonist may differ between molecularly distinct colon cancer cells. In this study, responses to the IAP antagonist Birinapant and oxaliplatin/5-fluorouracil (5-FU) were investigated in 14 colon cancer cell lines, representing the consensus molecular subtypes (CMS). Treatment with Birinapant alone did not result in a substantial increase in apoptotic cells in this cell line panel. Annexin-V/PI assays quantified by flow cytometry and high-content screening showed that Birinapant increased responses of CMS1 and partially CMS3 cell lines to oxaliplatin/5-FU, whereas CMS2 cells were not effectively sensitized. FRET-based imaging of caspase-8 and -3 activation validated these differences at the single-cell level, with CMS1 cells displaying sustained activation of caspase-8-like activity during Birinapant and oxaliplatin/5-FU co-treatment, ultimately activating the intrinsic mitochondrial apoptosis pathway. In CMS2 cell lines, Birinapant exhibited synergistic effects in combination with TNFα, suggesting that Birinapant can restore extrinsic apoptosis signaling in the context of inflammatory signals in this subtype. To explore this further, we co-cultured CMS2 and CMS1 colon cancer cells with peripheral blood mononuclear cells. We observed increased cell death during Birinapant single treatment in these co-cultures, which was abrogated by anti-TNFα-neutralizing antibodies. Collectively, our study demonstrates that IAP inhibition is a promising modulator of response to oxaliplatin/5-FU in colorectal cancers of the CMS1 subtype, and may show promise as in the CMS2 subtype, suggesting that molecular subtyping may aid as a patient stratification tool for IAP antagonists in this disease.

## Introduction

Colorectal cancer (CRC) is one of the leading causes of cancer-related deaths with the second-highest mortality rate of all cancers Worldwide^1^. High-risk stage II, as well as stage III and IV patients, are commonly treated with a combination of oxaliplatin, 5-fluorouracil (5-FU) and folinic acid (FOLFOX)^2,3^. However, the benefits of chemotherapy are highly variable, reflecting the inter-patient heterogeneity of colorectal cancers^4,5^. Analysis of the molecular basis of inter-patient heterogeneity is a critical first step in understanding why some patients benefit from specific treatments, while others fail to benefit. With the aim of understanding molecular differences between CRC tumors, several groups performed unsupervised cluster analysis of gene expression data coming up with several distinct but overlapping classifications^6–9^. In 2015, the CRC Subtyping Consortium combined six classifiers and defined four Consensus Molecular Subtypes (CMS) based on their transcriptomic profiles^10^: CMS1 represents microsatellite unstable tumors (MSI) with high levels of immune cell infiltration; CMS2 shows patterns of WNT and MYC pathway activation; CMS3 tumors are characterized by metabolic deregulation and *KRAS* mutations; and CMS4 is defined as the mesenchymal subtype, showing strong stromal infiltration and evidence of epithelial–mesenchymal transition (EMT)^10,11^.

The CMS classification system has been demonstrated to have some prognostic value, with CMS4 tumors showing the worst relapse-free and overall survival^10^. Whether the CMS classification has predictive value is still being debated, but it has been suggested that CMS2 patients in particular benefit from 5-FU-based chemotherapy^10,12–14^. Nevertheless, >20% of CMS2 and >30% of the tumors in all other subgroups will recur within 5 years, underlining the need for new therapies implemented in a targeted way to responsive patient subpopulations^12^. With the exception of CMS4, these CMS subtypes can be accurately represented by colon cancer cell lines making them a suitable platform to evaluate drug efficacy^15^.

Resistance to chemotherapy often results from defective apoptosis pathways^16,17^. Inhibitor of apoptosis proteins (IAPs) are important anti-apoptotic proteins that are overexpressed in various tumors; moreover, their overexpression often correlates with a poor prognosis^18–21^. IAPs can alter the downstream signaling after TNF receptor activation by inhibition of caspases and activation of the NF-κB pathway, thus transforming a potential death signal (TNFα) into a pro-survival signal^22^. In colorectal cancer, it was shown that patients with high expression levels of XIAP and cIAP2 have reduced disease-free survival and are more resistant to chemotherapy^21,23^. We previously showed that the expression levels of key apoptotic proteins, including XIAP, can successfully determine benefit from adjuvant chemotherapy and identify high-risk colorectal cancer patients^24–26^.

The second mitochondria-derived activator of caspases (SMAC) protein inhibits IAP function, and multiple small molecules mimicking SMAC function have been developed over the last two decades^22^. XIAP inhibition by SMAC mimetics facilitates caspase-3 activation, while inhibition of cIAPs allows the formation of the RIPK1-dependent caspase-8 activation platform that regulates cell death^27,28^. Therefore, SMAC mimetics represent a promising therapeutic option to sensitize apoptosis-resistant tumors to chemotherapy. However, the molecular heterogeneity of colorectal cancers opens up the question of whether some CMS subtypes may be more susceptible to IAP inhibition than others.

The SMAC mimetic Birinapant (TL32711) binds with high affinity to XIAP, cIAP1, and cIAP2 resulting in the inhibition of TNFα-mediated NF-κB activation and promotion of cell death^29^. Birinapant is currently being assessed in clinical trials. Initial results indicate only marginal single-agent activity; however, combinations with chemotherapy may be more effective^30^. Importantly, no clinical studies have assessed the efficacy of Birinapant in relation to tumor subtypes. With ongoing advancements in the development of IAP antagonists, it is becoming increasingly important to understand which patients might benefit from treatment with these agents. In this study, we used colon cancer cell lines representative of CMS subtypes to identify subtypes that benefit from Birinapant treatment.

## Materials and methods

### CMS classification of cell lines

This classification is based on a publicly available dataset from Medico et al.^31^, downloaded from GEO (GSE59857). For CMS subtypes, the newest version of Guinney’s classifier was used with Random Forest (RF) prediction for CMS1–4 subtypes. “RF predicted CMS” column is the model prediction and “RF nearest CMS” column shows the model prediction for the nearest subtype.

### Cell culture

HCT116, HCT116-p53−/−, LS513, LS1034, and DLD-1 were cultured in RPMI. For LIM1215 cells, RPMI was supplemented with 0.6 µg/ml human insulin (Sigma). For MDST8, HT-29, LoVo, and GP5D, DMEM was used. RKO, C106, and LS174T were cultured in EMEM. SW620 were cultured in Leibovitz’s L-15 medium. All cell culture media were supplemented with 10% FBS, 2 mM L-glutamine, 100 U/ml penicillin, and 100 µg/ml streptomycin. All cell lines have been authenticated at the beginning of the study and again within 4 months after completion of the experiments. All cell lines have been tested for mycoplasma contamination frequently.

### Western blot

Cells were seeded in six-well plates at a density of 500,000 cells per well, let adhere overnight, and treated with 2 µM oxaliplatin/10 µM 5-fluorouracil, 1 µM Birinapant, 10 ng/ml TNFα for 48 h. Proteins were isolated using RIPA buffer (140 mM NaCl, 10 mM Tris-HCl (pH 8.0), 1 mM EDTA, 1% Triton X-100, 0.1% sodium deoxycholate, 0.1% SDS) with freshly added protease, and phosphatase inhibitors (Sigma Aldrich). NuPAGE Bis-Tris gradient gels (4–12%) were used for electrophoresis, and proteins were blotted using a semi-dry transfer blotter (PowerBlotter, Invitrogen). Anti-cleaved caspase-3 (Cat. # 9661), anti-cleaved caspase-7 (Cat. # 9491), anti-cleaved caspase-8 (Cat. # 9496), and anti-XIAP (Cat. # 14334) antibodies were purchased from CST and used at 1:1000 dilutions. Anti-cIAP1 (diluted 1:500; Cat. # ab108361) and anti-cIAP2 (diluted 1:1000; Cat. # ab32059) antibodies were ordered from Abcam. An anti-actin antibody (1:2000; Sigma Aldrich, Cat. # A5441) was used as a loading control. HRP-labeled anti-mouse and anti-rabbit antibodies (1:5000; Merck, Cat. # AP124P and Cat. # AP132P) were used for detection of the primary antibodies. Images were taken using a Fuji LAS4000 imager and ECL substrate (Fisher Scientific Ireland).

### Flow cytometry-based detection of cell death using Annexin-V/PI

Cells were seeded at a density of 200,000 cells per well in a 12-well plate and were let adhere overnight. The adherent cells were then washed with PBS and treated with 2 µM oxaliplatin/10 µM 5-fluorouracil (medac GmbH), 1 µM Birinapant (TL32711, Active Biochem), 10 ng/ml TNFα (Enzo Life Sciences), or a combination of the drugs. After 48 h treatment, cells were detached using TrypLE Express (Thermo Fisher Scientific) washed with Annexin-V binding buffer (10 mM HEPES, 140 mM NaCl, 2.5 mM CaCl_2_, pH 7.4) and stained with Annexin-V-FITC (1:100, Biolegend). Stained cells were washed with Annexin-V binding buffer and stained with propidium iodide (1:500, Sigma Aldrich). Stained cells were analyzed using a BD LSRII flow cytometer with the BD FACSDiva software (Version 6, BD) and FlowJo (Version 10, FlowJo LLC). Doublet cells were excluded using the FSC-H and FSC-A, and 10,000 single cells were measured per technical replicate. All assays were performed in three independent biological replicates with three technical replicates.

### High-content screening

Cells were seeded onto black, glass-bottomed 96-well plates at appropriate densities, treated for 48 h and stained with FITC-tagged Annexin-V (BD-Biosciences), propidium iodide (Sigma Aldrich), and Hoechst stain (Invitrogen). Cell death was then quantified using an Array Scan XTI high-content microscope (Thermo Scientific).

### Live-cell time-lapse imaging and analysis of caspase activation kinetics in single cells

Cells were cultured in 6-well plates until they reached ~70% confluency, then transfected with 0.6 μg of plasmid DNA (CFP-IETD-Venus^32^) and 6 µl of Lipofectamine 2000 (Invitrogen) in 1 ml Opti-MEM at 37 °C with 5% CO_2_ for 4–12 h. To generate stable cell lines, transfected cells were cultured in the presence of 0.5–1 mg/ml G-418 for 1–2 weeks, and fluorescent clones were picked and expanded. Cells stably expressing CFP-IETD-Venus probe were plated at a density of 25,000 cells/compartment on sterile 35/10-mm glass-bottom dishes with four compartments (Greiner Bio-One GmbH, Stuttgart, Germany), and grown in RPMI for 24 h to let them attach to the surface. Next day, 30 nM tetramethylrhodamine-methyl ester (TMRM) was added into the medium, cells were covered with embryo-tested sterile mineral oil and mounted on an Axiovert 200 M inverted microscope equipped with a 40 × 1.3 NA objective (Carl Zeiss, Jena, Germany), a back-illuminated cooled EM CCD camera (Andor Ixon BV 887-DCS, Andor Technologies, Belfast, Northern Ireland), and a stage incubator set to 37 °C and 5% CO_2_ (Pecon, Erbach, Germany). One compartment in the dish left untreated as control, other three compartments were treated with 1 µM Birinapant, 2 µM oxaliplatin/10 µM 5-fluorouracil, and combination of the drugs. CFP, FRET, Venus, TMRM, and transmitted light images were recorded for at least ~48 h with 4 min interval. Protocols for automated image acquisition and stage positioning were generated using MetaMorph software (version 7.1, Molecular Devices, LLC, Sunnyvale, CA, USA). Image processing (background subtraction, median filtering, generation of masks, and ratiometric calculations) was carried out using Fiji/ImageJ (version 1.52i, Wayne Rasband, NIH, Bethesda, MD, USA)^33^. Single-cell kinetics of IETD cleavage were analyzed using ratiometric images generated by dividing CFP by FRET, then a region in the cytosol of the corresponding cell was drawn to measure the kinetics of TMRM.

### Co-cultures with peripheral blood mononuclear cells

HCT116, GP5D, LS513, and MDST8 cancer cell lines were seeded at a density of 5000 cells per well in a 12-well plate. Peripheral blood mononuclear cells (PBMCs) from healthy donors were isolated using Lymphoprep density-gradient medium (Stemcell Technologies) according to the manufacturer’s instructions. The blood donations were anonymous, and the donors have been randomly chosen. Isolated PBMCs were counted and 500,000 cells were seeded per well in a 12-well transwell insert with a membrane pore size of 0.4 µM (Corning). PBMCs were always cultured in RPMI supplemented with FBS, P/S, and L-glutamine independently of the co-cultured CRC cell line. The PBMCs were co-cultured overnight to let the monocytes adhere. The next day, the transwell inserts were washed with PBS to remove most floating immune cells. The cells were then co-cultured with the CRC cell lines for another 5 days to allow the monocytes to adapt to the cancer cells. After that incubation, the inserts were transferred to another 12-well plate, in which the CRC cells were seeded at a concentration of 200,000 cells per well. The cells were allowed to adapt for 1 day, and the treatment was started afterwards. For the treatment with TNFα-neutralizing antibodies, 500 ng/ml anti-TNFα antibodies (R&D Systems) were added to the respective treatment groups. Cells were analyzed as described before. All assays were performed in three independent biological replicates with two technical replicates.

### Light-sheet fluorescence microscopy

HCT116, GP5D, LS513, or MDST8 cells were seeded into individual wells of a 96-Well spheroid plate (96-well Black/Clear Round Bottom Ultra-Low Attachment Spheroid Microplate, Corning, UK) at a density of 1000 cells/well and grown in the medium as described earlier but supplemented with Hoechst 33258 (1 µg/ml) and PI (1 µg/ml) to stain for live-cell imaging (both Sigma Aldrich, Ireland) and without phenol red at 37 °C with 5% CO_2_ for 48–72 h—depending on cell type—until they formed spheres. The spheroids were then treated for 48 h, as indicated. For imaging, the spheroids were embedded in 1% low-melting agarose in PBS (Sigma Aldrich, Ireland) at 38–40 °C doped with sub-resolution beads at a concentration of 1:1000 of the original stock (PS-Speck, Thermo Fisher, Ireland), sucked into a glass capillary while liquid. When hardened, the capillary was mounted in the microscope sample holder and the chamber of the Light Sheet Fluorescence Microscope (Lightsheet Z1, Carl Zeiss, Germany) using glass capillaries with an inner diameter of 1.0 mm and an outer diameter of 1.5 mm. A plunger was used to push the agar with the embedded spheroids out of the capillary into the liquid in front of the 20 ×1.0 NA lens. The light sheet was generated with two 10 ×0.2 NA lenses illuminating the sample alternating from each side using the pivot scan mode. Images were taken at zoom 1.0 resulting in a light sheet thickness of 3.3 µm (405 nm excitation). Hoechst 33258 was excited using the 405-nm laser line, PI with 561 nm both using the 405/488/561-nm notch filter. To split the emission onto the two PCO edge sCMOS cameras filter cubes with beam splitter 510 nm using band-pass filters of 420–470 nm (Hoechst) and 575–615 nm (PI). Image stacks were then taken moving the object along the optical axis of the imaging objective at 1 µm steps and subsequently turning the object in 45° steps and re-aligning the object for each of the next 4 stacks. All images were processed using ZEN black and FiJi (ImageJ 1.52r-t). The multiview fusion and deconvolution were performed using the dedicated FiJi plugins (MultiView Reconstruction v5.0.20^34^) using the sub-resolution beads present in the agarose as an alignment aid for registration and for the generation of the PSF used for deconvolution. For the image fusion processing, the scale was set to half of the original scale using a server with dual Xeon Gold and 192 GB RAM (Power Edge R 740 XD, Dell EMC, Ireland), deconvolution was done with 5123-pixel block size. 3D projections processed from the resulting stacks are then presented in the figures.

### Transcriptomics data analysis

The gene expression patterns of TNFα, cIAP1, cIAP2, XIAP, and DIABLO by CMS group were investigated in two independent CRC patient cohorts: Taxonomy^14^ and The Cancer Genome Atlas (TCGA, restricted to the colon (COAD) and rectal (READ) cases). Transcriptomic profiles were downloaded from the Gene Expression Omnibus collection (GSE103479)^14^ and the TCGA PanCanAtlas release for the Taxonomy and the TCGA COAD-READ cohorts, respectively. For the Taxonomy cohort, probe-level measurements were collapsed to gene-level expression by mapping probe IDs from the Almac Diagnostics Custom Xcel array (GPL23985) to gene symbols and by aggregating multiple probes associated with the same gene by mean. For the TCGA COAD-READ cohort, the level 4 batch-normalized RNASeqV2 per-gene profiles were used as is without further processing. CMS labels were retrieved from files associated with the original publications^10,14^. Patients with available CMS labels (excluding “NOLBL”) and gene expression quantified in primary tumor tissue were retained for downstream analysis, totaling *n* = 632 cases (*n* = 135 and *n* = 497 for the Taxonomy and TCGA COAD-READ cohorts, respectively).

The association between expression of the selected genes and CMS classification for the patients form the Taxonomy (GSE103479)^14^ and TCGA COAD-READ cohorts were plotted and analyzed separately (as the data source, normalization, and units differ). For each marker, the expression was plotted as swarm plot stratified by CMS class. Outliers were defined as data-points more extreme than 1.5 times the interquartile range. When outliers were detected, the scale for the *y* axis uses a mixed linear/logarithmic scale. The transition between the linear and logarithmic range (for outliers) is marked with a dotted line. For plots that do not have extreme outliers, the *y* axis uses a standard linear scale. Association between marker expression levels (continuous, including outliers) and CMS classification were analyzed by regression models. Statistical significance was determined by overall ANOVA *P* values. Pairwise group comparisons were performed with Tukey HSD post hoc tests and mean expression difference, 95% confidence intervals (CI, lower and upper) and *P* values were reported (Supplementary File 2). *P* values were not adjusted for multiple comparisons, as these analyses were considered exploratory.

Visualizations and statistical analyses were performed using Python (version 3.7.5), including the libraries pandas (version 0.25.3), matplotlib (version 3.1.1), seaborn (version 0.9.0) and statsmodels (version 0.10.0).

### Ethics statement

PBMCs were donated by healthy volunteers with written consent according to the declaration of Helsinki. The blood sample collection was approved by the Research Ethics Committee of the Royal College of Surgeons in Ireland (REC1279).

### Statistics

Statistical analysis was performed using GraphPad Prism Software (Version 8, La Jolla, California, USA). Experimental results done in three biological replicates with three (Annexin-V/PI assay in single-cell culture) or two (PBMC co-culture experiments) technical replicates each. The mean of the technical replicates was used per biological replicate. A two-way ANOVA followed by Tukey’s test for multiple comparisons was used. The CMS4 subtype was excluded from the ANOVA analysis because only one cell line was in this group. For the comparison of treatment outcomes of co-culture experiments, multiple *t* tests were used. The Holm–Sidak method was used to correct for multiple testing and determination of statistical significance, *P < 0.05, **P < 0.01, ***P < 0.001.

## Results

We downloaded a publicly available dataset (GEO GSE59857) from Medico et al.^31^ and used the newest version of the CMSclassifier package with the Random Forest prediction method for CMS classification of colon cancer cell lines^10^. We then chose 14 cell lines covering all CMS subtypes. The selected cell lines and their genetic status are shown in Table 1. Importantly, the CMS4 subtype is strongly influenced and defined by the tumor microenvironment^10^; therefore, a cell culture setting might not be representative of this specific subtype. We highlight this limitation throughout the manuscript using quotation marks and only included one cell line for the “CMS4” group.

**Table 1:** Cell line panel used in the study.

	MSI/MSS status	KRAS status	BRAF status	PIK3CA status	PTEN status	P53 Status	Consensus molecular subtype	CRC intrinsic subtype
RKO	MSI	WT	V600E	H1047R	Positive	WT	CMS1	CRIS-D
HT-29	MSS	WT	V600E	P449T	Positive	R273H	CMS1	CRIS-B
HCT116	MSI	G13D	WT	H1047R	Positive	WT	CMS1	CRIS-D
HCT116-p53^−/−^	MSI	G13D	WT	H1047R	Positive	KO	CMS1	CRIS-E
LoVo	MSI	G13D	WT	WT	Positive	WT	CMS1	CRIS-B
GP5D	MSI	G12D	WT	H1047L	Positive	WT	CMS2	CRIS-C
LIM1215	MSI	WT	WT	WT	Positive	WT	CMS2	CRIS-C
LS1034	MSS	A146T	WT	WT	Positive	G245S	CMS2	CRIS-D
DLD-1	MSI	G13D	WT	E545K	Positive	S241F	CMS2	CRIS-A
SW620	MSS	G12V	WT	WT	Positive	R273H	CMS2	CRIS-D
C106	MSS	G13C	WT	WT	Positive	T125M	CMS2	CRIS-C
LS174T	MSI	G12D	WT	H1047R	Positive	WT	CMS3	CRIS-A
LS513	MSS	G12D	WT	WT	Positive	WT	CMS3	CRIS-E
MDST8	MSS	WT	V600K	WT	Null	WT	CMS4	CRIS-D

### Co-treatment with IAP antagonist leads to cIAP depletion and earlier and enhanced chemotherapy-induced caspase cleavage

**Fig. 1:**
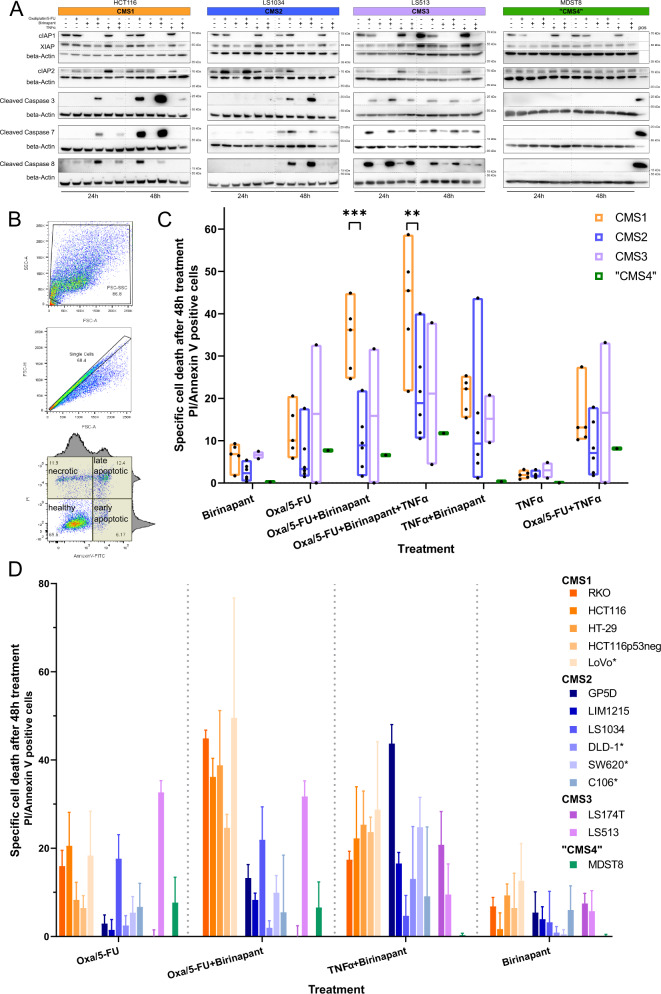
Response to Birinapant treatments in colon cancer cell lines. **A** HCT116, LS1034, LS513, and MDST8 cells were treated for 24 h and 48 h with the indicated treatments. The colored bars represent the subtype of the cell lines (CMS1—orange; CMS2—blue; CMS3—pink; CMS4—green). Blots were probed for cIAP1, cIAP2, and XIAP as well as the cleaved forms of caspase-3, -7, and -8. **B**–**D** All cell lines were treated for 48 h with 10 ng/ml TNFα, 1 µM Birinapant, 2 µM oxaliplatin/ 10 µM 5-FU (Oxa/5-FU) or a combination of these drugs. **B** Gating strategy for apoptosis assay. Only the sum of necrotic, early-, and late-apoptotic cells are shown in **C** and **D**. **C** Combined treatment results of all cell lines by CMS subtype. To normalize cell line intrinsic cell death, the background cell death (DMSO control) was subtracted. A two-way ANOVA was performed, excluding the CMS4 subtype. A Tukey’s test for multiple comparisons was used for post hoc analysis. **D** Treatment outcomes per cell line (mean + SD), grouped by their subtypes. To highlight the effects of Birinapant only, the single treatments and their corresponding co-treatment with Birinapant are shown. *N* = 3; *P ≤ 0.05; **P ≤ 0.01; ***P ≤ 0.001.

To assess whether targeting IAPs leads to cIAP degradation and cleavage and activation of downstream caspases, we first treated a selection of four colon cancer cell lines from each CMS subgroup, HCT116, LS1034, LS513, and MDST8 cells, with Birinapant and oxaliplatin/5-FU alone and in combination, and performed a western blotting analysis. Since Birinapant sensitizes cells to TNF receptor-induced apoptosis, we also co-treated the cells with TNFα, mimicking a pro-inflammatory tumor microenvironment (TME). We analyzed the protein levels of cIAP1, cIAP2, and XIAP as well as the activation of key caspases following the treatments. As expected, Birinapant effectively depleted cIAP1 (Fig. 1A, upper panel) in all treatment groups after 24 h and 48 h. Birinapant also led to a reduction of cIAP2 levels after 48 h in HCT116, LS1034, and LS513 cells. In MDST8 cells, however, cIAP2 was not depleted following Birinapant treatments. In line with the data published before^28,35^, XIAP was not degraded by Birinapant although we did see a slight reduction of XIAP levels in HCT116, LS1034, and LS513 cells when Birinapant was co-treated with either chemotherapy or TNFα after 48 h. Nevertheless, Birinapant single treatments did not activate downstream caspases in all cell lines, indicating that IAP antagonism alone was not sufficient to activate apoptosis. Oxaliplatin/5-FU treatments induced caspase-8 and caspase-3 activation after 24 h only in LS513 cells; however, caspase activation was also detected in response to oxaliplatin/5-FU treatment at 48 h in the HCT116 and LS1034 models. Co-treatments with Birinapant and oxaliplatin/5-FU led to cleavage of caspases-3, -7, and -8 in HCT116 and LS513 cells already after 24 h, indicating a more rapid activation of apoptosis signaling in these cells than in the chemotherapy alone group. TNFα/Birinapant co-treatments also induced caspase activation after 24 h in HCT116 and LS513 cells, although this was less pronounced compared to the FOLFOX co-treatments. In contrast, little to no caspase activation was observed in either LS1034 or MDST8 cells in response to TNFα/Birinapant; in fact, no caspase cleavage was observed after 24 h or 48 h in MDST8 cells in any treatment group. Collectively, these data indicate that Birinapant is on-target (depletion of cIAP1) and increases activation of caspases in response to oxaliplatin/5-FU in the HCT116 and LS1034 models. Furthermore, Birinapant enhanced sensitivity to TNFα-induced apoptosis in these models, albeit to a lesser extent than oxaliplatin/5-FU.

### CRC cell lines show CMS-specific treatment responses

We next quantified the cell death responses of each cell line in our selected panel (Table 1). Annexin-V/PI was used to quantify the apoptotic cells after 48 h (Fig. 1B). Birinapant alone had minimal effect on apoptosis in all cell lines (Fig. 1C, D). Treatment with oxaliplatin/5-FU, which mimics the current clinical standard-of-care treatment, modestly increased cell death in most CRC cell lines, with only one (LS513) exhibiting an increase of over 30% at this time point (Fig. 1D, light pink bar). Except for the LS1034 cells, CMS2 cell lines did not respond to oxaliplatin/5-FU (Fig. 1C, D). The “CMS4” cell line MDST8 also exhibited only modest apoptotic sensitivity to oxaliplatin/5-FU.

Co-treatments with Birinapant and oxaliplatin/5-FU revealed some CMS-specific responses, with a significant increase in apoptotic cells in all five CMS1 cell lines (Fig. 1C, D). Two of the six CMS2 cell lines (GP5D and LIM1215) showed a modest increase in cell death in response to oxaliplatin/5-FU/Birinapant co-treatments, whereas the remaining four cell lines showed no increase compared to oxaliplatin/5-FU alone. In the CMS3 models and the “CMS4” MDST8 model, the addition of Birinapant did not increase the observed cell death. Hence, our results showed that CMS1 tumor models were the most sensitive to apoptosis induced by Birinapant co-treatment with oxaliplatin/5-FU.

In the context of co-incubation with TNFα, Birinapant activated apoptosis in all but the MDST8 model (Fig. 1C, D). In CMS1 cells treated with TNFα/Birinapant, the observed cell death was less than in response to co-treatment with oxaliplatin/5-FU and Birinapant. However, the synergistic effects of TNFα and Birinapant were especially pronounced in the CMS2 cell lines LIM1215, GP5D, and SW620 cells. In addition, both CMS3 cell lines in our panel had an increased amount of apoptotic cells following the treatment with TNFα/Birinapant.

Co-incubation of oxaliplatin/5-FU/Birinapant-treated cells with TNFα resulted in the highest cell death in most models, but only significantly increased the cell death in three cell lines (HCT116-p53^−/−^, HT-29, and LIM1215) compared to the most effective double treatment. As expected based on our previous publications, the lack of p53 in HCT116 cells resulted in generally higher resistance to oxaliplatin/5-FU treatments. However, this resistance was overcome following the addition of Birinapant to oxaliplatin/5-FU in the context of co-incubation with TNFα.

**Fig. 2:**
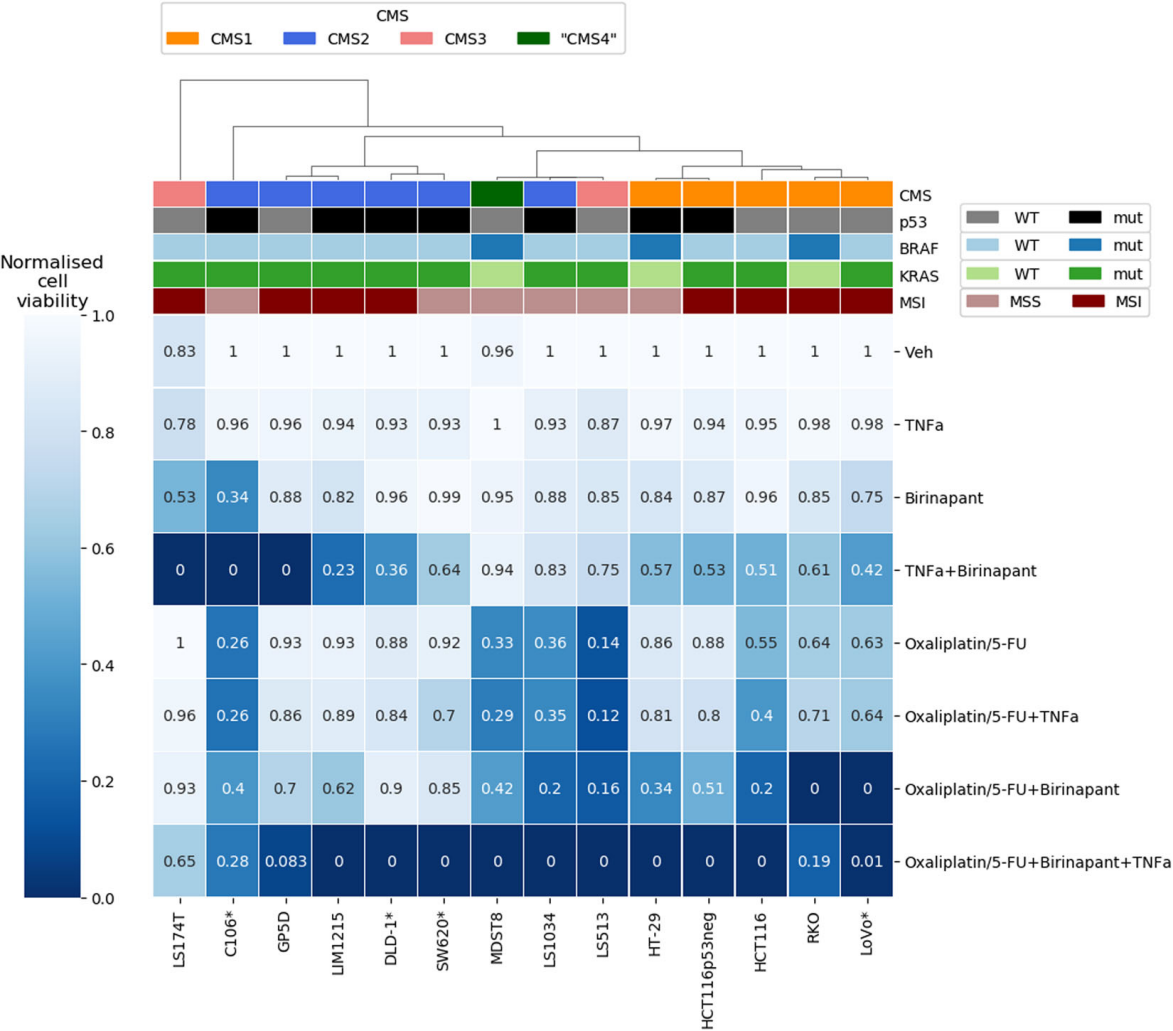
Cluster analysis of treatment outcomes. A hierarchically clustered heatmap was generated using the complete Euclidean distance method with normalized amount of cell viability after 48 h treatment. The first row represents the CMS of the respective cell lines and the KRAS, BRAF, p53 mutational status as well as the microsatellite stability (MSS/MSI) of the cell lines. For cell lines labeled with an asterisk, viability was assessed by an Annexin-V/PI assay using high-content-screening. For all other cell lines, viability was determined by flow cytometry.

To further investigate differences and similarities of the treatment outcomes, we performed an unsupervised hierarchical clustering to elucidate common response patterns between the cell lines and subtypes. Since we observed a high degree of variability in the cell viability, we normalized the viability (amount of healthy cells) after 48 h by subtracting the minimum viability of all treatment groups and dividing it by the maximum viability. Thus, for each cell line, the highest observed viability was assigned the number one and the lowest viability was assigned zero, independently of background cell death or cell line-specific differences. We found three general treatment response clusters (Fig. 2). All CMS1 cell lines formed a cluster that showed the strongest responses to oxaliplatin/5-FU and Birinapant co-treatments. CMS2 cell lines, except the LS1034 cell line, formed a separate cluster with strong responses to TNFα and Birinapant co-treatments. The third cluster, consisting of MDST8, LS1034, and LS513 cell lines, represents chemo-sensitive cell lines, which did not show strong benefits of Birinapant co-treatments. Neither *BRAF*, *KRAS*, and *Tp53* mutations nor the microsatellite instability (MSI) was predictive for these clusters or the (co-)treatment responses.

Collectively, these results suggest that CMS1 cells respond best to oxaliplatin/5-FU treatment in combination with Birinapant, whereas CMS2 cell lines respond best to Birinapant/TNFα co-treatments.

### Single-cell time-lapse analysis reveals CMS-specific caspase activation

**Fig. 3:**
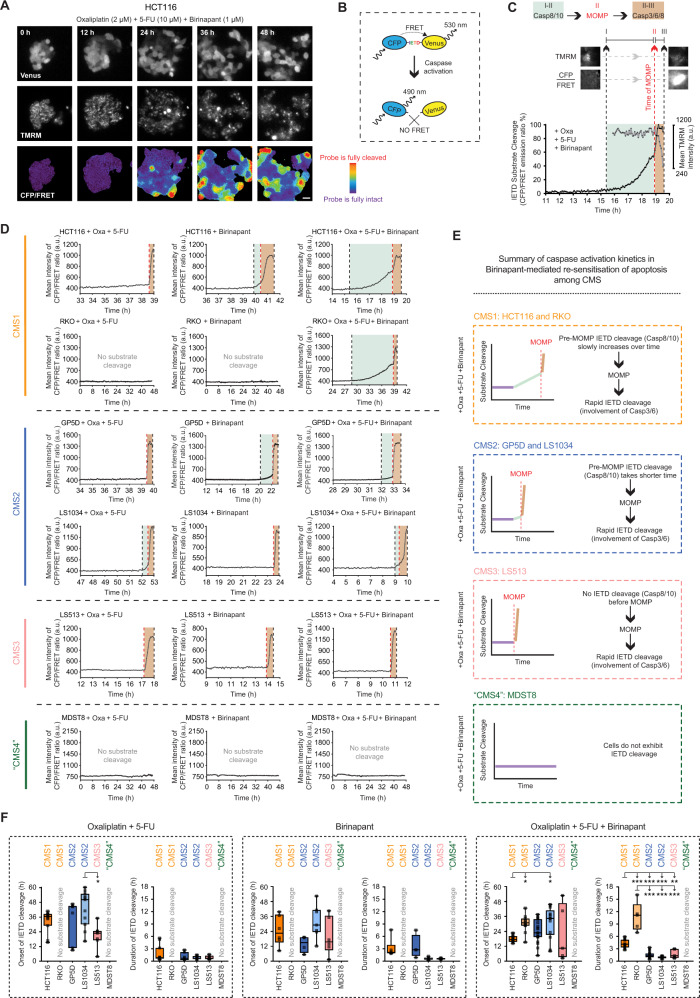
CMS subtypes show distinct differences in caspase activation kinetics in response to co-treatment of Birinapant and chemotherapy. **A**, **B** Representative time-lapse images of Venus, TMRM and CFP/FRET emission ratio in HCT116 (**A**) cells stably expressing IETD-FRET probe (**B**) treated with 2 µM oxaliplatin/10 µM 5-FU/1 µM Birinapant for 48 h. Scale bar: 20 µm. **C** Overview of generation and interpretation of a single-cell trace extracted from time-lapse imaging of IETD-FRET probe cleavage in an HCT116 cell showing features of apoptosis in response to combination treatment. Cleavage of IETD substrate by caspases results in a disruption in FRET signal, which can be quantified by measuring CFP/FRET emission ratio. A mild increase in CFP/FRET emission ratio (I) before the loss of TMRM signal due to MOMP (II, red dashed line) indicates caspase-8-like activity (I–II, color coded as light blue). A rapid increase in CFP/FRET emission ratio (III) after the loss of TMRM signal indicates the involvement of executioner caspases (caspase-3 and -6) (II–III, color coded as light brown). **D**, **E** Representative single-cell CFP/FRET emission ratio kinetics of HCT116 (CMS1), RKO (CMS1), GP5D (CMS2), LS1034 (CMS2), LS513 (CMS3), and MDST8 (CMS4) cell lines treated with Oxa/5-FU, Birinapant, or combination of the drugs (**D**). Summary of the differences in caspase activation kinetics among CMS in response to co-treatment of Oxa/5-FU and Birinapant (**E**). **F** Quantification and comparison of the differences in caspase activation kinetics among CMS in response to Oxa/5-FU (left), Birinapant (middle), or combination of the drugs (right). “Onset of IETD cleavage” indicates a point of time at which cells start cleaving IETD substrate, “Duration of IETD cleavage” refers to a period of time between onset and end of IETD cleavage. Data are shown as individual values for each cell as well as the median ± quartiles. *n* = 146 cells were analyzed from at least three experiments per cell line. Statistical significance was tested using one-way ANOVA followed by Tukey’s multiple comparison test. “no substrate cleavage” indicates that cells did not exhibit an increase in IETD cleavage over the course of the experiment, *P ≤ 0.05; **P ≤ 0.01; ***P ≤ 0.001.

To investigate apoptosis signaling in real-time in the different CMS models, we measured the kinetics of caspase activation in response to oxaliplatin/5-FU and Birinapant, alone or in combination, by single-cell time-lapse microscopy in HCT116 and RKO (CMS1), GP5D and LS1034 (CMS2), LS513 (CMS3) and MDST8 (“CMS4”) cell lines. We stably transfected the cells with CFP-IETD-Venus FRET probe (Fig. 3A), in which CFP and Venus fluorophores are linked with Ile–Glu–Thr–Asp (IETD) sequence that shows high selectivity for caspase-8 and 10 ^32,36^, but is also cleavable by caspase-3 and -6^37^ (Fig. 3B). Detecting mitochondrial membrane potential depolarization by TMRM simultaneously with FRET measurement allows for accurate differentiation of initiation and execution phases during apoptosis^32,38^. A typical apoptotic cell undergoing death receptor-induced apoptosis exhibits slowly increasing IETD cleavage indicating caspase-8 activation (Fig. 3C, I–II), followed by a drop in TMRM intensity due to MOMP (Fig. 3C, II) and lastly a rapid increase in post-MOMP IETD cleavage indicating the involvement of executioner caspases (e.g., caspase-3/-6) (Fig. 3C, II–III). In contrast, cells undergoing BCL-2 family-controlled mitochondrial apoptosis typically do not show significant IETD cleavage prior to MOMP^32,39,40^.

In line with previous findings^28^, we observed two noticeable IETD cleavage patterns in treated and responding cells. In the first type, cells did not exhibit pre-MOMP IETD cleavage, but a drop in TMRM intensity followed by a rapid increase in IETD cleavage was still observable, suggesting that apoptosis was induced by direct activation of the mitochondrial or intrinsic pathway. This type of pattern was generally evident in response to chemotherapy in HCT116, GP5D, and LS513 and to Birinapant in LS513 and LS1034. The second type of IETD cleavage pattern involved pre-MOMP IETD cleavage followed by a drop in TMRM intensity and a rapid increase in IETD cleavage as seen in response to Birinapant in HCT116 and GP5D and in response to oxaliplatin/5-FU in LS1034 (Fig. 3D).^28^

Notably, single-cell analysis of IETD-FRET measurements in cells treated with Birinapant in combination with oxaliplatin/5-FU revealed CMS-specific caspase activation patterns (Fig. 3D, E). In HCT116 and RKO cell lines (CMS1), we observed a moderate increase in pre-MOMP IETD cleavage that persisted for hours (3.7 ± 1.68 h in HCT116 and 11.2 ± 4.17 h in RKO). Then there was a drop in TMRM intensity, followed by a rapid increase in IETD cleavage, suggesting that both extrinsic and intrinsic apoptotic pathways were activated. In LS1034 and GP5D cell lines (CMS2), we observed either a slight (0.6 ± 0.21 h for LS1034, 1.17 ± 0.32 h for GP5D) or no increase in pre-MOMP IETD cleavage, followed by a drop in TMRM intensity and a rapid increase in IETD cleavage. LS513 (CMS3) cells did not display pre-MOMP IETD cleavage, but a drop in TMRM intensity and post-MOMP IETD cleavage was evident, suggesting that apoptosis was induced by rapid activation of the intrinsic pathway. In MDST8 cell line (“CMS4”), cells did not exhibit any increase in IETD cleavage during the time of the experiment (Fig. 3D, right panels), consistent with results presented in Fig. 1.

We then compiled IETD-FRET measurements under two main features, “Onset of IETD cleavage” and “Duration of IETD cleavage” (Fig. 3F and Supplementary File 1). “Onset of IETD cleavage” indicates a point of time at which individual cells start exhibiting IETD cleavage (“I” in Fig. 3C); and “Duration of IETD cleavage” refers to a period of time between onset and end of IETD cleavage (“I–III” in Fig. 3C). LS513 cells showed a significantly earlier apoptotic response to oxaliplatin/5-FU compared to LS1034 (Fig. 3F, left). However, the duration of IETD cleavage did not differ between CMS subtypes. In response to Birinapant, there was no difference in the onset and duration of IETD cleavage between groups (Fig. 3F, middle). Strikingly, in response to co-treatment with Birinapant and oxaliplatin/5-FU, duration of IETD cleavage was significantly longer in HCT116 and RKO (CMS1) cells compared to GP5D, LS1034, and LS513 cells. Moreover, we observed that IETD cleavage in HCT116 cells started earlier compared to LS1034. Taken together, our results suggest CMS-specific differences in real-time apoptosis activation kinetics. Moreover, the synergistic interaction between Birinapant and chemotherapy in CMS1 cell lines can be mechanistically explained by efficient induction of apoptosis involving activation of both extrinsic and intrinsic signaling pathways.

### Birinapant efficacy is enhanced by healthy immune cells in CMS1 and CMS2 cells

**Fig. 4:**
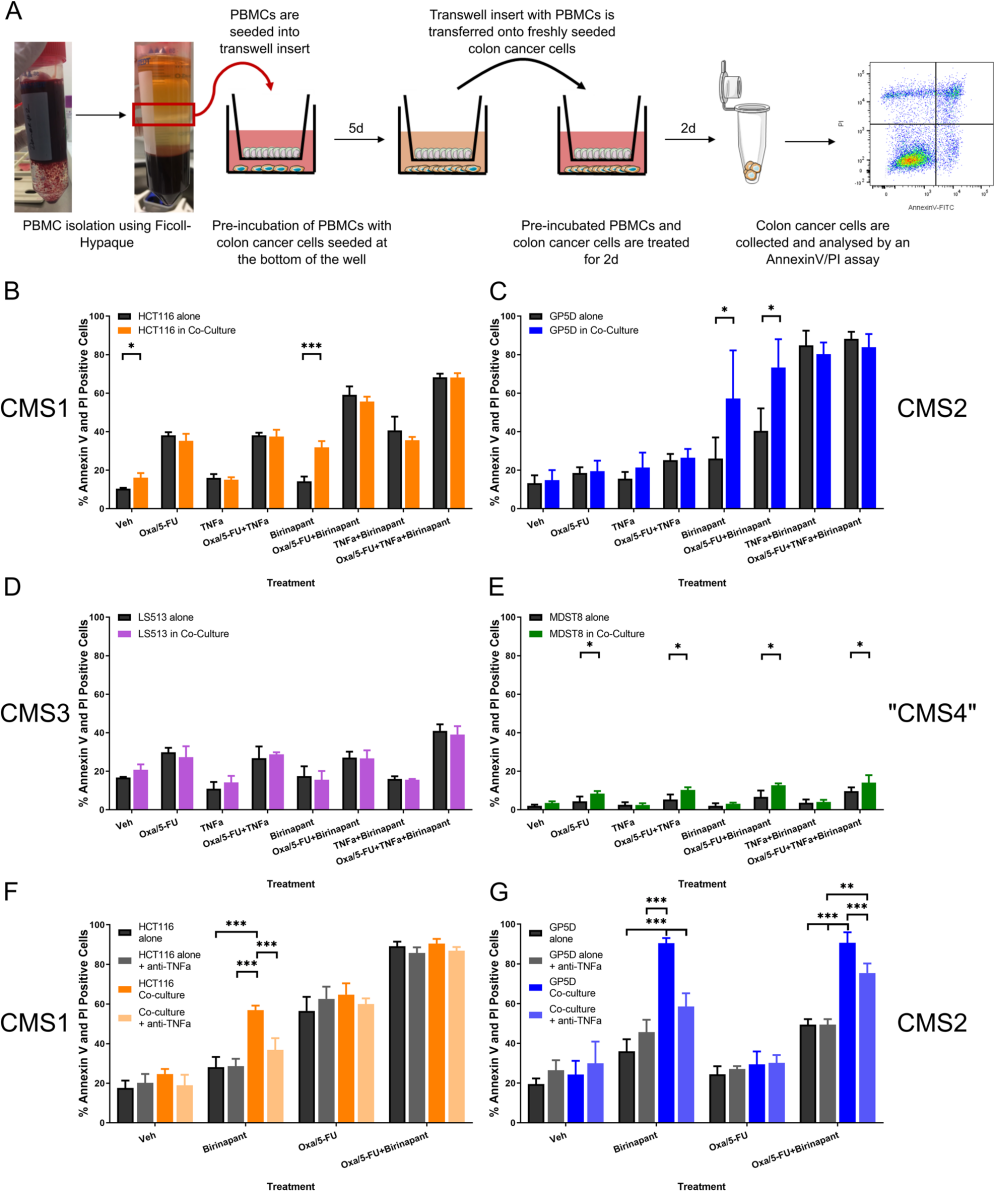
Co-culture of colon cancer cell lines with healthy PBMCs. **A** Overview of the workflow. PBMCs were isolated using Ficoll-Hypaque density centrifugation, transferred in a cell culture transwell insert (0.4-µm pore size) and incubated for 5 days with the respective CRC cells. After 5 days, the insert with the PBMCs was transferred into a new well with newly seeded CRC cells. Following one day of co-culture, the cells were treated with 2 µM oxaliplatin/10 µM 5-FU/1 µM Birinapant for 2 days and analysed using an Annexin-V/PI assay. **B**–**E** Amount of apoptotic and necrotic cells after 48 h treatment in the absence (black) or presence (colored bar) of immune cells. The subtypes are represented by the colored bar with the CMS1 cell line HCT116 represented in orange (**B**), the CMS2 cell line GP5D in blue (**C**), CMS3 in pink (**D**, LS513), and the “CMS4” cell line MDST8 in green (**E**). Additionally, HCT116 (**F**) and GP5D (**G**) were co-incubated with TNFα-neutralizing antibodies during the treatments in co-culture. Significance was determined by multiple *t* tests using the Holm–Sidak method for multiple comparisons. *N* = 3; *P ≤ 0.05; **P ≤ 0.01; ***P ≤ 0.001.

Since TNFα is just one component of a typical TME, we next assessed how co-cultured immune cells influenced Birinapant treatment responses in the different subtypes. The co-cultured PBMCs were plated into cell culture inserts with a pore size of 0.4 µM, thus allowing the exchange of media between the compartments but keeping the cells separated. The impact of peripheral blood mononuclear cells (PBMCs) on apoptosis of colon cancer cells treated with oxaliplatin/5-FU and Birinapant alone and in combination was investigated (Fig. 4A). We found that HCT116 (CMS1) and GP5D (CMS2) cells responded significantly better to Birinapant single treatment in co-culture with PBMCs (Fig. 4B, C). The FOLFOX-sensitive HCT116 cells did not show an additional increase in cell death in co-culture during FOLFOX/Birinapant co-treatments, whereas GP5D cells showed a significant increase in apoptosis after chemotherapy with Birinapant treatments (Fig. 4C). The highly resistant MDST8 cell line had a significantly increased cell death in the co-cultures with PBMCs in response to both oxaliplatin/5-FU single and co-treatments, although absolute levels of cell death remained low (Fig. 4E). No differences in the sensitivity of the CMS3 cell line LS513 between control and co-culture were observed (Fig. 4D). The addition of anti-TNFα-antibodies almost completely abrogated apoptosis induction in response to Birinapant single treatment in co-cultured HCT116 and GP5D cells (Fig. 4F, G), indicating that this effect was driven by TNFα secreted by the PBMCs. However, TNFα-neutralizing antibodies only partially abrogated the effects of the FOLFOX/Birinapant combination in PBMC co-cultured GP5D cells (Fig. 4G), suggesting that factors other than TNFα might be important in the context of co-treatment with FOLFOX. Collectively, these results show that the paracrine secretion of TNFα by immune cells increases the efficacy of Birinapant as a single agent and in combination with chemotherapy.

### CMS-specific responses are also found in 3D-models

**Fig. 5:**
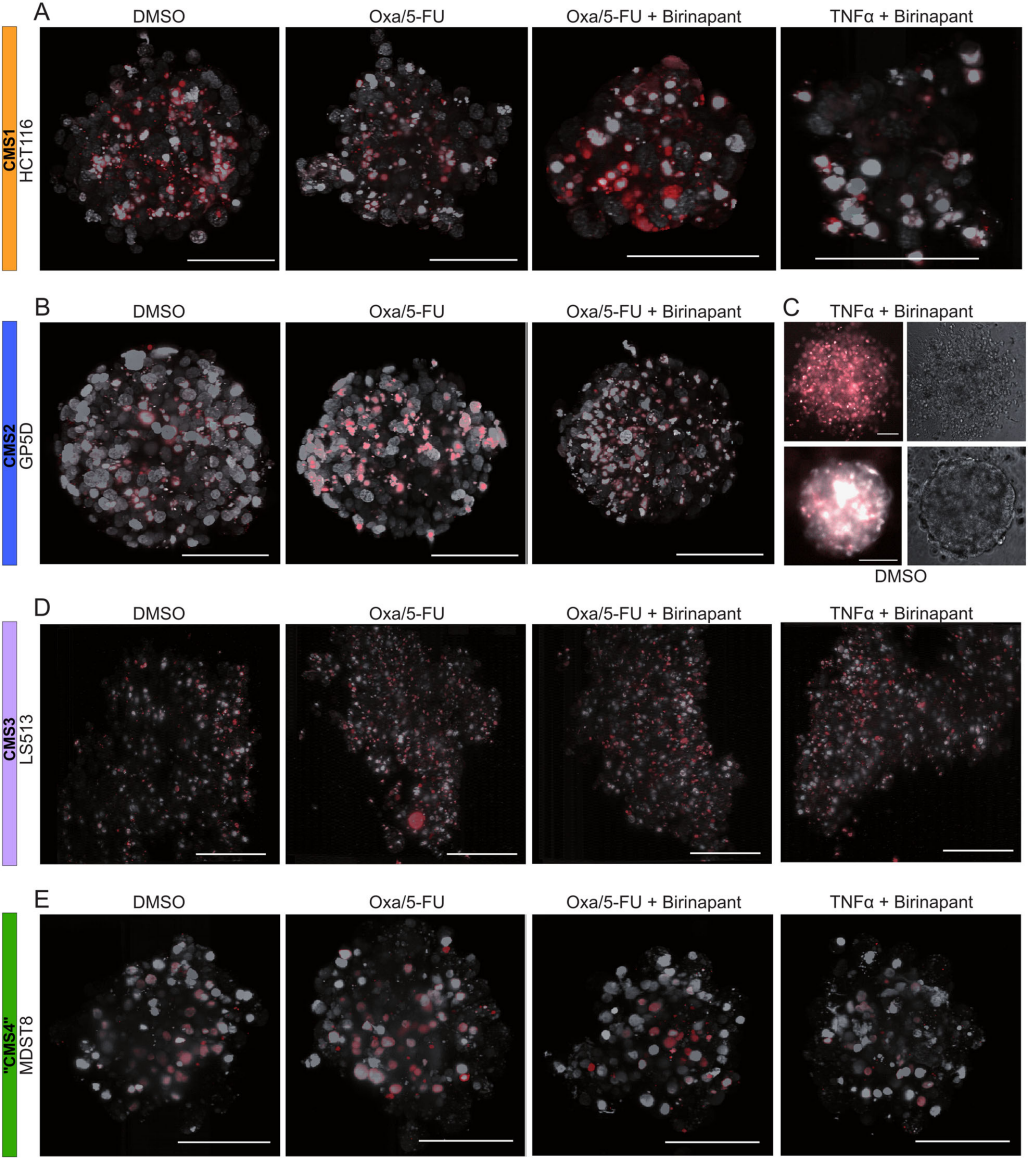
Light-sheet microscopy images of representative cell lines grown in spheroids. HCT116 (**A**), GP5D (**B**, **C**), LS513 (**D**), and MDST8 (**E**) cells were grown in 3D spheroids and treated with the indicated treatments for 48 h, Hoechst-stained nuclei are displayed in grayscale and PI-stained in red. **C** shows an image taken on an inverted microscope with a 10× 0.3 NA objective and CCD camera and appropriate filters set for Hoechst and PI (Nikon TE 300 with Lumencor Sola II 365 and SPOT RT) as the spheroid broke-up following treatment and it was not possible to mount the debris for light-sheet fluorescence microscopy. The scale bar always indicates a length of 100 µm in the object space. Shown are representative images of three individual repeats.

To better mimic the complex interaction of the tumor cells and to assess its influence on the treatment responses, we grew four selected cell lines in three-dimensional spheroids and treated them for 48 h (Fig. 5). In line with our data from the 2D models, HCT116 spheroids (CMS1) showed an increased amount of apoptotic cells following oxaliplatin/5-FU/Birinapant co-treatments (Fig. 5A). We did not observe an increase of dead cells with the TNFα/Birinapant treatment, but the spheroids were generally smaller than the controls. GP5D spheroids (CMS2), in turn, showed an increase of apoptotic cells with the oxaliplatin/5-FU treatment, but we did not see additional cell death following the addition of Birinapant (Fig. 5B). However, with TNFα/Birinapant treatments, we saw extensive cell death which led to the complete disintegration of the spheroid (Fig. 5C). Similar to our findings with the 2D cultures, we found an increase of apoptotic cells with oxaliplatin/5-FU treatments in our CMS3 model (Fig. 5D), which is not further increased by the addition of Birinapant. MDST8 spheroids (“CMS4”) showed only minimal apoptosis during the treatments and Birinapant did not induce further cell death (Fig. 5E). Again, these results show that Birinapant in combination with chemotherapy was most effective in our CMS1 models and that Birinapant successfully sensitized CMS2 cells to TNFα-induced cell death.

### TNFα expression differs between tumor subtypes

**Fig. 6:**
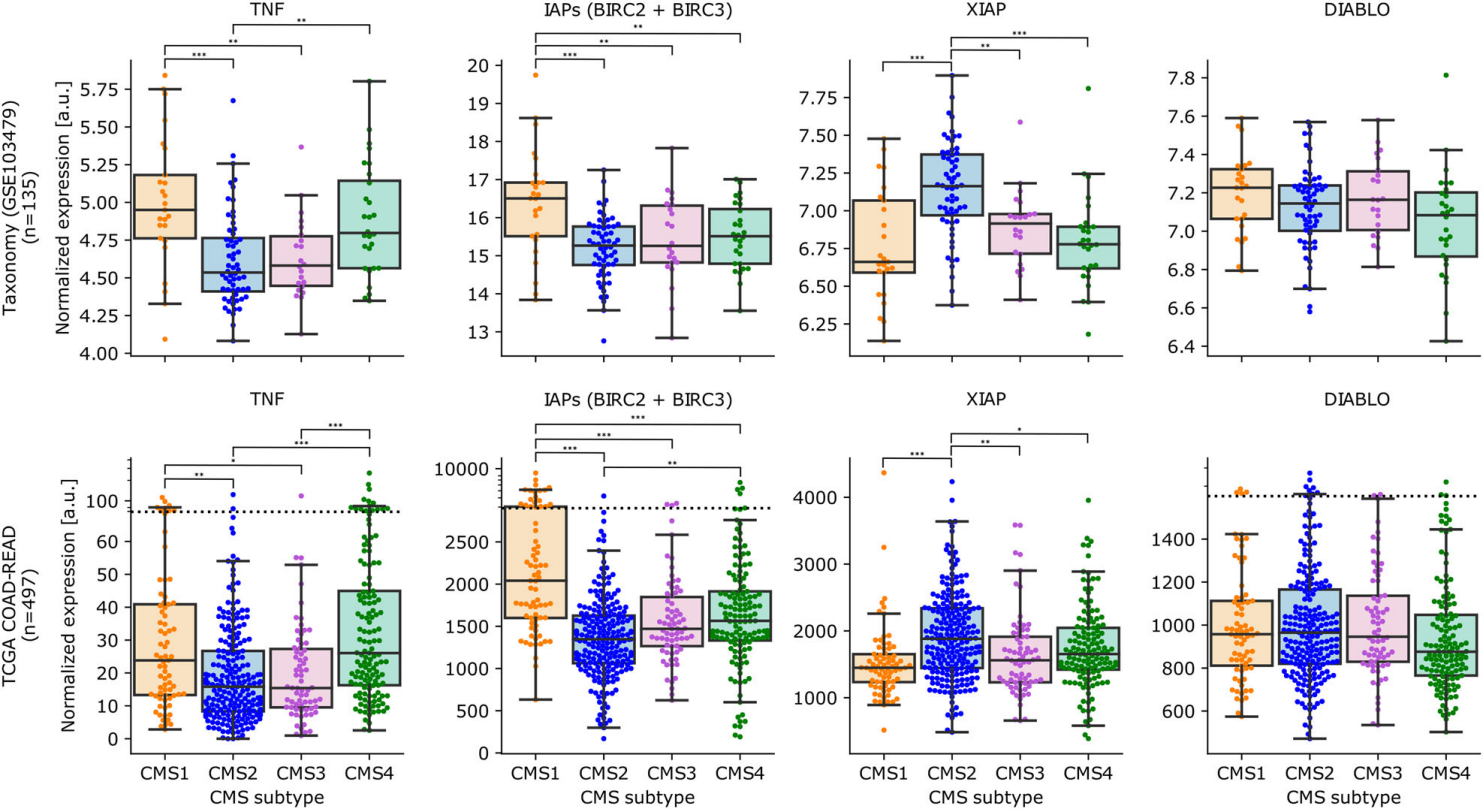
Gene expression profiles of key players involved in Birinapant adjuvant therapy from primary tumor tissue exhibit CMS subtype-specific patterns. Two independent CRC cohorts (Taxonomy and TCGA COAD-READ) were analyzed. Dotted line indicates a transition from linear to logarithmic space in the presence of outliers (see “Materials and methods”). Statistical analysis can be found in Supplementary File 2. Pairwise group comparisons were performed with Tukey HSD post hoc tests. *P* values were not adjusted for multiple comparisons as these analyses were considered exploratory. *P ≤ 0.05; **P ≤ 0.01; ***P ≤ 0.001.

Having shown that the TNFα secretion of the TME is an essential factor for the efficacy of Birinapant, we analyzed microarray data of our cohort of stage 2/3 CRC patients (GSE103479)^14^ and used the COAD TCGA dataset to validate our findings. We then assessed the expression levels of TNFα, cIAP1 (BIRC2), cIAP2 (BIRC3), XIAP, and DIABLO in the tumors (Fig. 6). In both datasets, CMS1 and CMS4 tumors showed the highest levels of TNFα expression, whereas CMS2 and CMS3 tumors showed significantly lower levels. Moreover, CMS1 samples also showed significantly higher expression of cIAPs (BIRC2 and BIRC3) compared to the other subtypes. Furthermore, in the TCGA dataset, CMS2 tumors also have significantly lower cIAP1 and 2 expressions compared to CMS4 tumors. In turn, CMS2 tumors showed an upregulation of XIAP expression in the TCGA and Taxonomy cohort. Lastly, the expression levels of DIABLO (SMAC) did not show any significant differences between the subtypes (statistics and individual figures can be found in Supplementary File 2 and Supplementary Fig. 1).

## Discussion

Reliable patient stratification remains a major challenge in treating CRC, and it was recently shown that patients with stage 2/3 CMS2 and stage 3 CMS1 tumors benefit the most from the FOLFOX treatment, whereas CMS4 tumors do not benefit from adjuvant chemotherapy^12,41^. In this study, we systematically assessed the efficacy of Birinapant treatment in combination with the standard-of-care treatment FOLFOX using colon cancer cell lines from each CMS subgroup, although the lack of stroma likely changes the behavior of the mesenchymal CMS4 subtype.

In line with data published before^28,42,43^, Birinapant showed very limited efficacy as a single agent. However, we found a subtype-dependent response to co-treatments. In CMS1 cell lines in particular and also CMS2, Birinapant increased the efficacy of the standard-of-care oxaliplatin/5-FU combination chemotherapy regimen. We reasoned that the differential responses of specific CMS subtypes might be explained by CMS-specific differences during apoptosis execution. Birinapant-mediated restoration of efficient apoptosis is primarily dependent on cIAP1/2-dependent activation of caspase-8 at the ripoptosome complex rather than XIAP-dependent regulation of executioner caspases^28,44^. CMS1 cell lines exhibited a slow caspase-8 activation whereas CMS2 and CMS3 cell lines either displayed very brief caspase-8 activation prior MOMP or caspase activation after MOMP. Similar patterns in response to Birinapant alone or oxaliplatin/5-FU were reported in a previous study^28^. Heterogeneity in caspase activation kinetics during apoptosis can arise from several sources such as cell-to-cell variability in mitochondrial content^45^ or expression levels of proteins from the BCL-2-family^46^, p53^47^, and c-FLIP^48^. Paek et al.^47^ showed that chemotherapy-induced, p53-dependent upregulation of IAPs prevented efficient execution of apoptosis in HCT116 (CMS1) cells. Further corroborating our findings, they demonstrated that combining chemotherapy with an IAP inhibitor successfully restored both caspase-8-dependent and -independent apoptosis in HCT116 cells^47^.

Since the tumor microenvironment is a crucial determinant for disease progression and treatment outcomes^49^, we mimicked immune infiltration using co-cultures of cancer cells and PBMCs. We showed that in the presence of immune cells, Birinapant could improve cell death even as a single agent in CMS1 and CMS2 cells in a manner dependent on immune cell-derived TNFα. MSI tumors of the immune CMS1 subtype already have a high number of infiltrating tumor cells and thus, are susceptible to immunotherapy which was approved by the FDA in 2017^50,51^. The high expression levels of TNFα in combination with high cIAP expression levels also make CMS1 tumors the most promising subtype for Birinapant-containing regimens. CMS2 tumors, however, generally have much less immune infiltration, and the immune cells are often exhausted, thus unable to control tumor growth^52–54^. These tumors are considered “immune-cold” with low levels of immune infiltration and, in line with that, we found only low TNFα expression in CMS2 tumors. In this study, we only focused on the paracrine secretion of cytokines, mainly TNFα, by immune cells and their direct effects on the tumor cells. It has been shown before, however, that IAP antagonists can also co-stimulate effector T-cells and NK cells, thus furthering the potential clinical impact of IAP antagonists^55–57^. In fact, a clinical trial combining Pembrolizumab and Birinapant is currently recruiting (ClinicalTrials.gov: NCT02587962). Enhanced activity of immune effector cells in CMS2 immune-cold tumors (and indeed immune-hot CMS1 tumors) would not only enhance TNFα levels, but also levels of immune effector cell-associated death ligands FasL/CD95L and TRAIL in the tumor microenvironment, the anti-tumor activity of which is also enhanced by IAP antagonists^58–61^. Thus, as well as the IAP antagonist-mediated enhancement of chemotherapy-induced apoptosis in the tumor compartment of CMS1 and CMS2 cancers, an additional anti-tumor activity would be expected through modulation of the tumor immune microenvironment.

In conclusion, our study provides the first proof-of-concept that the differential apoptosis susceptibilities of CMS subtypes are a determinant of the efficacy of direct (and most likely indirect) cell death-targeting therapeutics. Moreover, our studies indicate that CMS1 and CMS2 are the subtypes that are most likely to be responsive to treatment with an IAP antagonist.

## Supplementary information

Supplementary Figure Legends

Supplementary Figure 1

Supplementary Figure 2

Supplementary Figure 3

Supplementary File 1

Supplementary File 2

Supplementary Methods

## Data Availability

Data and analysis code are publicly available and archived at Zenodo at 10.5281/zenodo.3630638.
