# Supplementary material for: Molecular subtype-specific responses of colon cancer cells to the SMAC mimetic Birinapant
Source: Cell Death Dis. 2020 Nov 30;11(11):1020. doi: 10.1038/s41419-020-03232-z (PMC7705699; doi:10.1038/s41419-020-03232-z)
Supplement: Supplementary file 1 — Supplementary Figure Legends [file 41419_2020_3232_MOESM1_ESM.docx]

**Supplementary Figure 1: Gene expression profiles of cIAP1 (BIRC2) and cIAP2 (BIRC3).**

Two independent CRC cohorts (Taxonomy and TCGA COAD-READ) were analyzed. Dotted line indicates transition from linear to logarithmic space in the presence of outliers. Statistical analysis can be found in Supplementary File 3.

**Supplementary Figure 2: Treatment responses after 24h, 48h and 72h.**

All cell lines were treated with 10 µM 5-FU/2 µM Oxaliplatin, 1 µM Birinapant, 10 ng/ml TNFα or a combination for the indicated time. Cell death was assessed by Annexin-V/PI assay using high-content screening (HCS).

**Supplementary Figure 3: Synergy assays with representative cell lines.**

HCT116, GP5D, LS513 and MDST8 cell lines were treated with a combination of Oxaliplatin/5-FU+Birinapant or TNFα+Birinapant. The viability was assessed using a MTT assay and the synergy scores were calculated.

**Supplementary File 1: Raw data of IETD-FRET measurements.**

**Supplementary File 2: Statistical analysis of gene expression profiles.**
