# Supplementary figures and images for: Molecular subtype-specific responses of colon cancer cells to the SMAC mimetic Birinapant
Source: Cell Death Dis. 2020 Nov 30;11(11):1020. doi: 10.1038/s41419-020-03232-z (PMC7705699; doi:10.1038/s41419-020-03232-z)

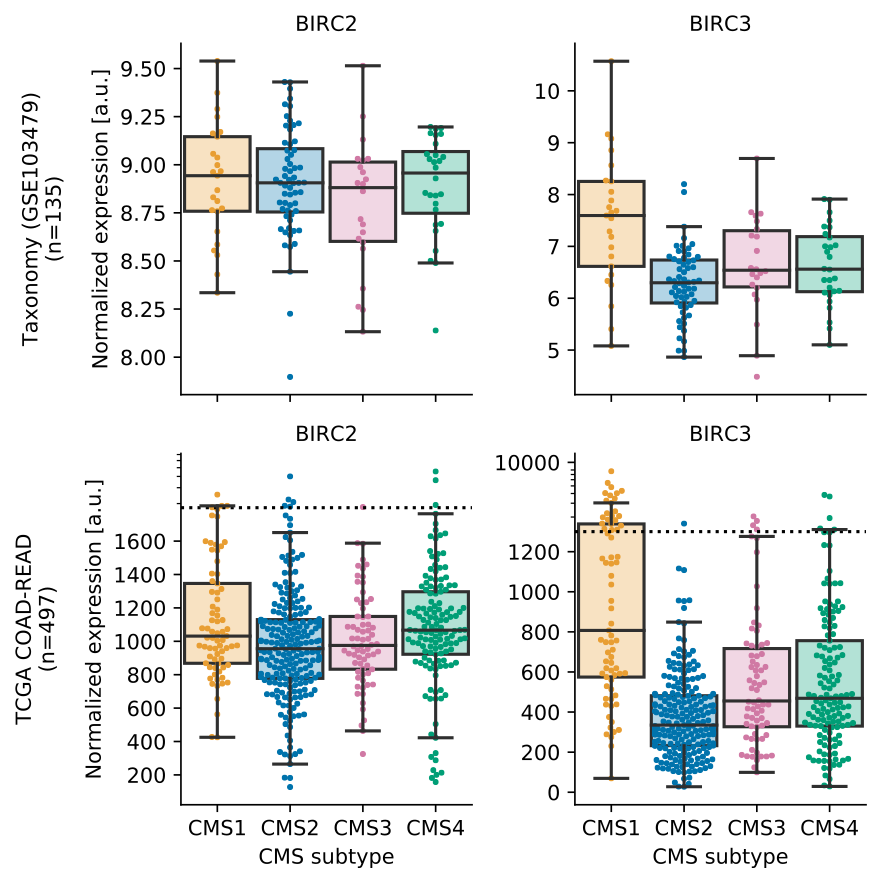

Supplement: Supplementary file 2 — Supplementary Figure 1 [file 41419_2020_3232_MOESM2_ESM.png]

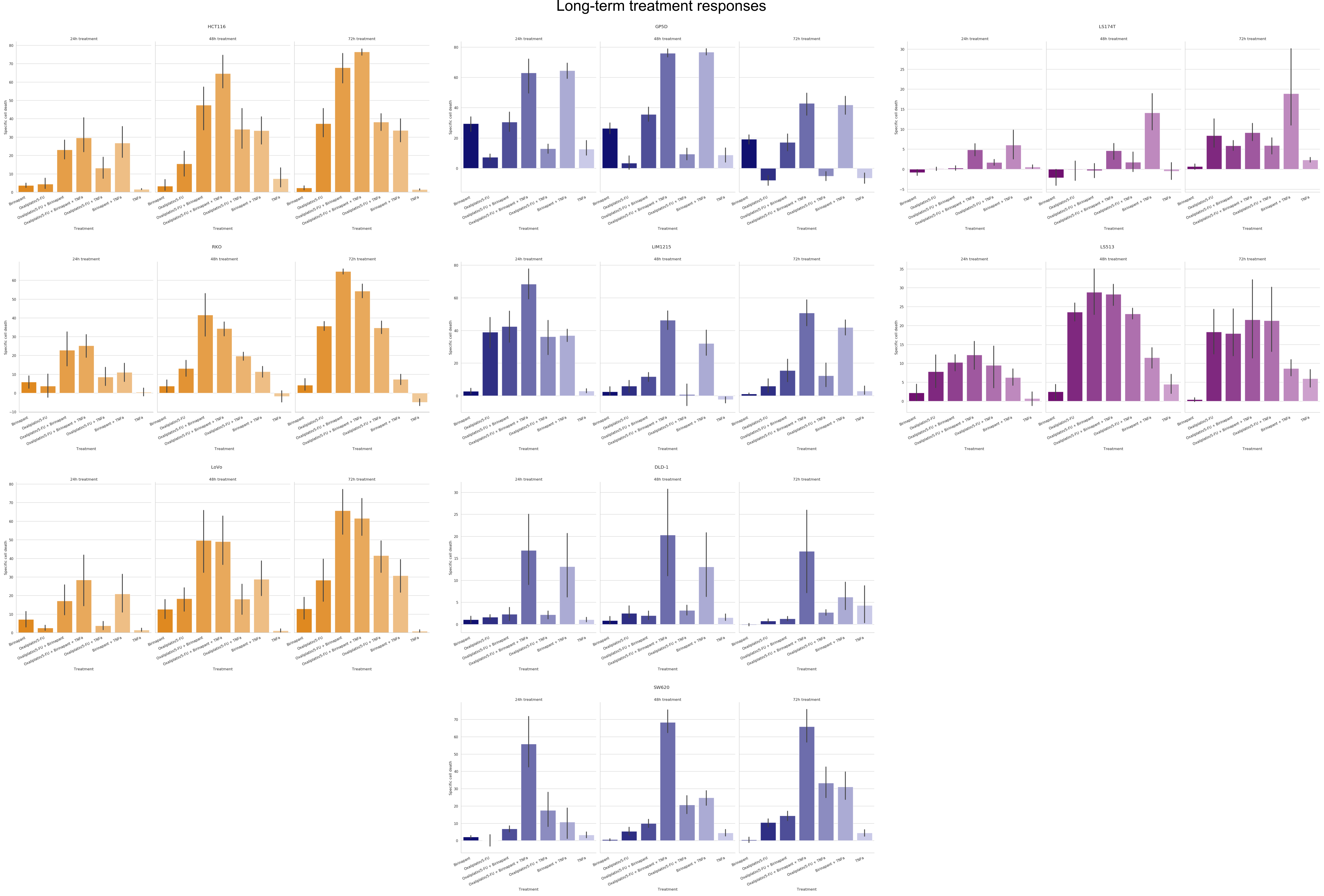

Supplement: Supplementary file 3 — Supplementary Figure 2 [file 41419_2020_3232_MOESM3_ESM.png]

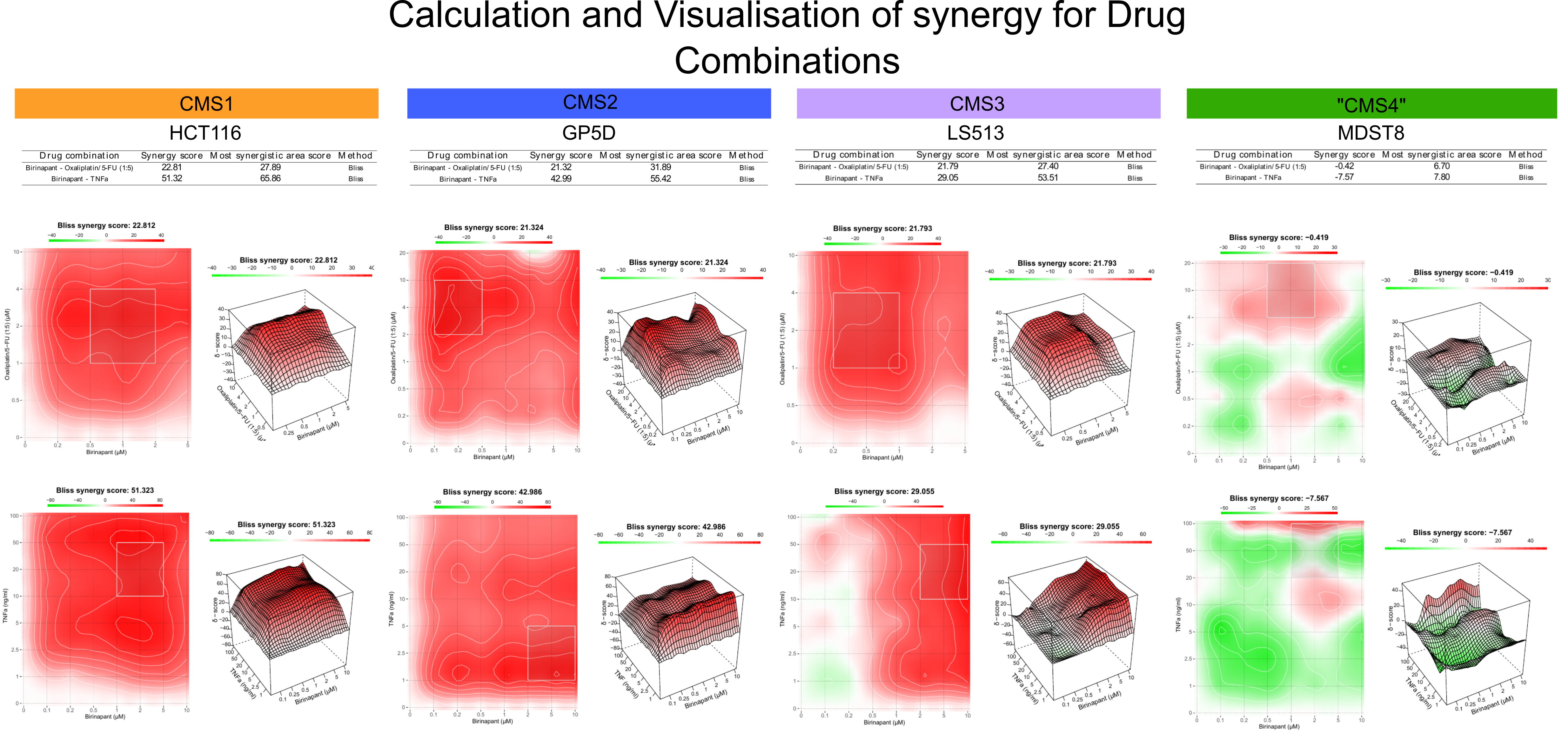

Supplement: Supplementary file 4 — Supplementary Figure 3 [file 41419_2020_3232_MOESM4_ESM.png]
