# Supplementary material for: Molecular subtype-specific responses of colon cancer cells to the SMAC mimetic Birinapant
Source: Cell Death Dis. 2020 Nov 30;11(11):1020. doi: 10.1038/s41419-020-03232-z (PMC7705699; doi:10.1038/s41419-020-03232-z)
Supplement: Supplementary file 7 — Supplementary Methods [file 41419_2020_3232_MOESM7_ESM.docx]

**Synergy testing**

For each cell line, 5000 cells were seeded per well in a 96-well plate and incubated over night. For each well, separate dilutions were prepared, added onto the cells and the cells were then incubated for 48h. Afterwards, the medium was carefully removed, the cells were washed with PBS, and 100 ul acid phosphatase buffer (0.1 M sodium acetate, 0.1% Triton-X-100, 5 mM pNPP) was added to the cells. After 2h incubation at 37 °C in the dark, 50 ul 1M NaOH was added to each well, incubated for 5min and the absorbance was read at 405nm and 620nm wavelength on a CLARIOStar microplate reader (BMG labtech). Each experiment was repeated at least 4 times. The SynergyFinder 2.0 web application was used to calculate the synergy scores and to generate the figures (Supplementary Figure 3).
